# Comment on “Role of increased plasminogen activator inhibitor-1 and vitronectin in gestational diabetes mellitus”

**DOI:** 10.1590/1806-9282.20231607

**Published:** 2024-05-03

**Authors:** Hejia Yin, Rou Shi

**Affiliations:** 1 First Affiliated Hospital of Kunming Medical University, Department of Endocrinology II – Kunming, China.

Dear Editor,

Ozgen et al^1^. recently conducted a study that focused on exploring the role of increased plasminogen activator inhibitor-1 (PAI-1) and vitronectin in gestational diabetes mellitus (GDM). GDM is a condition characterized by elevated blood sugar levels during pregnancy and is associated with various health risks. The study aimed to investigate the involvement of two key factors, namely, PAI-1 and vitronectin, in the pathophysiology of GDM. The research involved the analysis of blood samples and clinical data from pregnant women with and without GDM to compare the levels of PAI-1 and vitronectin. In this study, it was found that levels of vitronectin and PAI-1 were significantly elevated in the GDM group when compared to the control group. The findings of the study may have provided insights into the mechanisms underlying GDM, particularly the potential contribution of these two factors to the development and progression of the condition. Understanding the role of increased PAI-1 and vitronectin in GDM could have significant implications for the diagnosis, treatment, and management of this condition. It might open up avenues for targeted therapies or interventions aimed at mitigating the adverse effects of GDM and improving maternal and fetal health during pregnancy. However, some of the concerns outlined below require further clarification and explanation.

First, it is important to emphasize that this study^1^ lacks a completely healthy-control group. Although this study included 60 women between 24 and 27/6 weeks of gestation as a control group, this evidence is insufficient because it is unclear how PAI-1 and vitronectin levels vary in a completely healthy-control group. Possible hypotheses are that in a completely healthy-control group, PAI-1 and vitronectin levels are at extremely high levels, and pregnancy or pregnancy combined with GDM results in a significant decrease in PAI-1 and vitronectin. Alternatively, PAI-1 and vitronectin levels may be extremely low in a completely healthy-control group, and pregnancy or pregnancy combined with GDM may lead to a significant increase in PAI-1 and vitronectin. Therefore, in the absence of a completely healthy-control group, the trends in changes in PAI-1 and vitronectin are not yet clear. Therefore, it is recommended to include a group of individuals who are age and body mass index-matched and do not have diabetes or pregnancy as a healthy-control group to further elucidate the trends in PAI-1 and vitronectin changes.

Second, the broad application of antenatal corticosteroids (ACS)^2,3^ in obstetrics is primarily driven by their proven efficacy in reducing the incidence and severity of critical conditions such as respiratory distress syndrome, intraventricular hemorrhage, necrotizing enterocolitis, and neonatal mortality associated with preterm births. Adding to this, emerging evidence^4^ also points to potential advantages of ACS administration for pregnant individuals diagnosed with GDM. It is worth highlighting that the study consisted of GDM patients, and among them, there exists the possibility that some received ACS treatment, leading to a substantial elevation in PAI-1 and vitronectin levels. Nevertheless, the study omitted any specific details regarding whether ACS therapy was administered to the participants. In the event that ACS was indeed given to the study subjects, the conspicuous increase in PAI-1 and vitronectin levels might be more attributable to ACS use rather than the presence of GDM itself. Hence, it remains imperative to furnish a comprehensive account of ACS utilization within the study population. Therefore, it is advisable to provide a detailed account of whether the included patients received ACS to mitigate potential confounding bias.
